# Human papillomavirus genotype distribution and socio-behavioural characteristics in women with cervical pre-cancer and cancer at the start of a human papillomavirus vaccination programme: the CIN3+ plus study

**DOI:** 10.1186/s12885-018-5248-y

**Published:** 2019-01-30

**Authors:** Dianne Egli-Gany, Anne Spaar Zographos, Joachim Diebold, Virginie Masserey Spicher, Brigitte Frey Tirri, Rolf Heusser, Joakim Dillner, Patrick Petignat, Roland Sahli, Nicola Low, on behalf of the CIN3+plus study group, Astrid Baege, Fridolin Bannwart, Clauldia Berlin, Pascal Cassinotti, Gieri Cathomas, Johanna Claass, Carina Eklund, Milo Frattini, Anne Graber, Anina Häfliger, Liza Ho, Andre Kind, Christian Kreis, Luca Mazzucchelli, Thomas McKee, Eric Megevand, Ulrike Meyer-Hamme, Francesca Molinari, Christoph Noppen, Aurelia Noske, Ines Raineri, Elisabetta Rapiti, Dominique Rubi, Niels J. Rupp, Kathrin Schwedler, Thomas Stallmach, Jean-Christophe Tille, Luigi Tornillo, Alexandra Valera, Dominique Weintraub, Philip Went, Dieter Zimmermann

**Affiliations:** 1 Institute of Social and Preventive Medicine, University of Bern, Mittelstrasse 43, 3012 Bern, Switzerland; 2 Federal Office of Public Health, Schwarzenburgstrasse 157, 3003 Bern, Switzerland; 3 Institute of Pathology, Cantonal Hospital Lucerne, Spitalstrasse, 6000 Luzern 16, Switzerland; 4 Department of Gynaecology and Obstetrics, Kantonsspital Baselland, Rheinstrasse 26, 4410 Liestal, Switzerland; 5 National Institute for Cancer Epidemiology and Registration, Hirschengraben 82, 8001 Zurich, Switzerland; 6 WHO HPV LabNet Global Reference Laboratory, Department of Clinical Microbiology, Center for Cervical Cancer Prevention, Skåne University Hospital, Malmö, Karolinska Institute and Hospital, SE-171, 77 Stockholm, Sweden; 7 Department of Gynaecology and Obstetrics, Geneva University Hospitals, Boulevard de la Cluse 30, 1205 Genève, Switzerland; 8 Institute of Microbiology, Lausanne University Hospital and University of Lausanne, Rue du Bugnon 48, 1011 Lausanne, Switzerland

**Keywords:** HPV, Human papillomavirus, Vaccines, Cervical cancer, Cervical dysplasia, Cervical intraepithelial neoplasia

## Abstract

**Background:**

The Swiss Federal Office of Public Health has recommended vaccination against human papillomavirus (HPV) to prevent cervical cancer since 2008. To establish monitoring of the future public health impact of vaccination, baseline population-based data are required. The objectives of this study were to examine the distribution of oncogenic HPV genotypes in biopsies with cervical intraepithelial neoplasia stage 3 or more severe lesions (CIN3+) at the beginning of HPV vaccination programmes and to compare sociodemographic and behavioural factors of women with CIN3+ with women in the Swiss general population.

**Methods:**

We conducted a retrospective and prospective cross-sectional study with women diagnosed with CIN3+ in Switzerland. Ten pathology institutes from six cantons and three language regions participated. We conducted HPV typing on formaldehyde fixed-paraffin embedded specimens from 2014 and 2015. Women enrolled in 2015 were asked to complete a questionnaire. We described frequencies of HPV types. We also compared demographic characteristics and socioeconomic status in the CIN3 + plus group with the Swiss National Cohort in 2014 and compared risk factors for HPV infection with the Swiss Health Survey in 2012.

**Results:**

We included 768 biopsies from 767 women. Four hundred and seventy-five (61.8%) biopsies were positive for HPV 16 and/or 18, 687 (89.5%) were positive for oncogenic HPV genotypes 16, 18, 31, 33, 45, 52, and/or 58 and five (0.7%) were HPV negative. Twenty-eight (10.3%) of the 273 women who completed the patient questionnaire reported having received at least one dose of an HPV vaccine. When compared with Swiss women in the six study cantons, fewer women in the CIN3+ plus study group were of Swiss nationality, more were born abroad and more were single. The study group also had a higher proportion of women with ≥2 partners in the last year, current smokers and was younger at age of first sexual intercourse.

**Conclusions:**

Introduction of the nonavalent vaccine could cover approximately 90% of CIN3+ lesions in Swiss women compared with around 60% with the quadrivalent vaccine. Surveillance of HPV genotype distribution in CIN3+, together with information about vaccination and CIN3+ incidence will allow monitoring of the public health impact of vaccination programmes.

**Trial Registration:**

ClinicalTrials.gov, NCT02323997. Registered 24 December 2014.

**Electronic supplementary material:**

The online version of this article (10.1186/s12885-018-5248-y) contains supplementary material, which is available to authorized users.

## Background

Human papillomavirus (HPV) vaccines provide strong protection against persistent HPV infections. Most HPV infections of the cervix are cleared by the immune system, but persistent HPV infections with some genotypes cause cervical cancer [1]. Of more than 100 identified HPV genotypes, the International Agency for Cancer Research (IARC) classifies 12 HPV genotypes (16, 18, 31, 33, 35, 39, 45, 51, 52, 56, 58 and 59) as causal agents of cervical cancer and another eight (26, 53, 66, 67, 68, 70, 73 and 82) as probable or possible causes of cervical cancer [2]. In a meta-analysis of studies from 61 countries worldwide, HPV types 16 and 18 were found in 70% of all invasive cervical cancer and 52% of high-grade squamous intraepithelial specimens [3]. Three types of HPV vaccines are licensed worldwide. Bivalent vaccines contain non-infectious virus-like particles (VLPs) of oncogenic HPV genotypes 16 and 18 [4, 5], quadrivalent vaccines contain VLPs of HPV 16 and 18 and HPV 6 and 11, which cause genital warts, and a nonavalent vaccine contains VLPs of HPV 16, 18, 31, 33, 45, 52 and 58 and types 6 and 11 [6]. All three vaccines have been approved for use in the European Union since 2015 [7] and the United States of America since 2014 [8] but, as of August 2018, only bivalent and quadrivalent vaccines are available in Switzerland. HPV vaccine has been introduced into vaccination programmes in 73 countries [9].

The real-world impact of HPV vaccination programmes on morbidity and mortality can only be evaluated by post-licensure comparison of HPV-associated outcomes before and after HPV vaccine introduction. The World Health Organization (WHO), European Centre for Disease Prevention and Control (ECDC) and Australian government [10] recommend monitoring of HPV infection prevalence, incidence of genital warts, incidence of pre-cancerous cervical intraepithelial neoplasia (CIN), and invasive cervical cancer incidence and mortality [11–13]. Cervical cancer takes up to 20 years to develop [14] and is rare in high income countries, so specimens collected over many years are needed to obtain sufficient precision to estimate HPV-type specific prevalence [10]. In Switzerland, descriptive data are limited. To assess HPV type distribution over a shorter time frame, a pragmatic disease outcome could include severe CIN (grade 3) or adenocarcinoma in situ, which usually develop seven to 15 years after HPV infection [15]; together with invasive cancer these conditions are referred to as CIN3+ [16].

We designed the CIN3+ plus study to provide information about the feasibility of a future surveillance system and the impact of HPV vaccination in Switzerland. In Switzerland, HPV vaccination programmes for young girls and women began in 2008, organised by health authorities in the 26 cantons (states) and targeting 11 to 14 year old girls, with recommendations for catch up vaccination for women up to 26 years of age. The Swiss Federal Office of Public Health conducted a national survey in 2014 with 2363 women aged 18–24 years and found that 41.3% (95% CI 36.4–46.3) reported receiving three HPV vaccine doses, 4.7% (95 CI 3.3–6-6) two doses and 5.4% (95% CI 3.6–8.0) one dose [17]. Cantonal cancer registries record incidence and mortality of cervical cancers, but not all record CIN3 and none records the HPV type [18]. The primary objective of this study was to determine the baseline distribution of oncogenic HPV genotypes in women diagnosed with CIN3+ in women living in Switzerland at the start of the cantonal vaccination programmes. Secondary objectives were to compare sociodemographic and epidemiological characteristics between women with CIN3+ and women in the general Swiss population.

## Methods

The CIN3+ plus study period was January 2014 to December 2015. We conducted a cross-sectional study in the year 2015, consisting of retrospectively analysed formaldehyde-fixed paraffin-embedded (FFPE) specimens from 2014 and samples and data from women enrolled prospectively during 2015. We assumed that the majority of women diagnosed with CIN3+ during the study period would not have received HPV vaccination, either because the target age group for vaccination would be too young to have developed CIN3+, or because the uptake of HPV vaccination amongst older women was too low to have had an impact on CIN3+ incidence. Ten pathology institutes from six cantons (Basel-City, Basel-Land, Geneva, Lucerne, Ticino and Zurich) and three language regions (German, French and Italian) participated in the study. Three institutes were based at publicly funded university hospitals, three were publicly funded non-academic institutes and four were private institutes. To select specimens collected in 2014, we provided the institutes with lists of random numbers, which they used to label biopsy specimens with a diagnosis of CIN3+ from women living in any of the participating cantons. We calculated the number of biopsies for each institute, based on the proportion of the total number of CIN3+ cases that each laboratory diagnosed in 2014. We used open access software (OpenEpi, version 3.1) to generate the random number lists. If there was not enough biopsy material for testing, we asked institutes to use the next biopsy with CIN3+. We enrolled women prospectively during 2015. We sent information about the CIN3+ plus study to gynaecologists of women who were diagnosed with CIN3+ and who met the inclusion criteria and asked them to obtain written informed consent from the woman. Inclusion criteria were: ≥18 years of age, living in one of the participating cantons and literate in German, French, Italian or English. We published a short description of the study methods and basic results in the bulletin of the Swiss Federal Office of Public Health [19].

### HPV genotyping

We conducted HPV typing on specimens prepared from stored FFPE blocks from cervical biopsies, cone biopsies, endometrial curettage specimens and/or hysterectomies. If more than one biopsy was available for a patient, we analysed the most recent and/or best preserved biopsy. Each institute conducted HPV typing according to their routine practice and standard operating procedures for deparaffinisation, tissue digestion and DNA extraction. All institutes used their routine nucleic acid amplification test, all of which included targets for the IARC-defined high-risk HPV genotypes 16, 18, 31, 33, 35, 39, 45, 51, 52, 56, 58 and 59 and a variety of other HPV genotypes (Additional file 1: Table S1). For specimens that tested negative for HPV, two aliquots of the purified DNA were sent to the WHO HPV Laboratory Network Reference Laboratory in Stockholm, Sweden [20] and tested using MGP-PCR followed by Luminex and B-globin real-time PCR. Their laboratory methods are described in a separate publication [21]. In addition, all participating laboratories were requested to participate in the WHO HPV Laboratory Network proficiency panel testing, a quality assurance system for HPV genotyping [20].

### Sociodemographic and behavioural data

We collected basic demographic data and clinical and histopathological diagnosis for all patients. Prospectively enrolled patients from 2015 who gave written informed consent were asked to complete a written questionnaire that asked for additional information, including HPV vaccination status, sociodemographic characteristics, smoking history and sexual behaviour. We entered all data into a piloted Research Electronic Data Capture form (REDCap™ Software, Vanderbilt University, Nashville, Tennessee, USA). We calculated a proxy of socioeconomic position (SEP) for all patients with a valid address using the Swiss neighbourhood index of SEP (Swiss-SEP) [22]. The Swiss-SEP is a small area-based measure based on median rent per square metre, proportion of households headed by a person with primary education or less, proportion of households headed by a person in a manual or unskilled occupation and the mean number of persons per room [22]. The Swiss-SEP index was developed with 2000 Swiss census data and has a range from zero (lowest SEP) to 100 (highest SEP), with a median of 63.32. When calculating the Swiss-SEP for our study, we used the closest residential house from the 2000 census to the geographic coordinate of interest. For comparison, we obtained demographic data from women in the general Swiss population from the Swiss National Cohort (SNC) [23] and behavioural data from the Swiss Health Survey (SHS) [24]. The SNC is a census-based cohort of basic individual-level data (e.g. canton, age, sex, etc.), which includes the entire Swiss population. Additional variables (e.g. education level, occupation) were collected with a structured written questionnaire, which was completed by a random subset of the Swiss population in 2014. The SHS is a written questionnaire and telephone interview conducted with randomly selected individuals living in Switzerland in 2012. Women ≥18 years of age living in one of the participating cantons were included from the SNC and SHS for analysis. An additional inclusion criterion for the SHS was that both the telephone interview and written questionnaire were completed.

### Sample size and statistical analysis

We planned a sample size of approximately 900 samples. The inclusion of samples from 2014 allowed us to increase the precision of estimates of type-specific HPV prevalence. The sample size was based on the number of CIN3+ cases diagnosed per year (approximately 760 per year in the participating cantons), a prevalence of 70% for HPV 16/18 [25] and an estimated response rate of 75%. We planned to test 250–500 retrospective biopsies from 2014 and 250–500 prospective biopsies from 2015. We performed analyses using statistical software (STATA, version 14.0, Statcorp, College Station, Texas USA). We applied age-adjusted weights to the SNC and SHS datasets to compare characteristics with women in the CIN3 + plus study group. We used chi-squared tests to compare nominal and ordinal variables and linear regression to compare continuous variables.

## Results

**Fig. 1:**
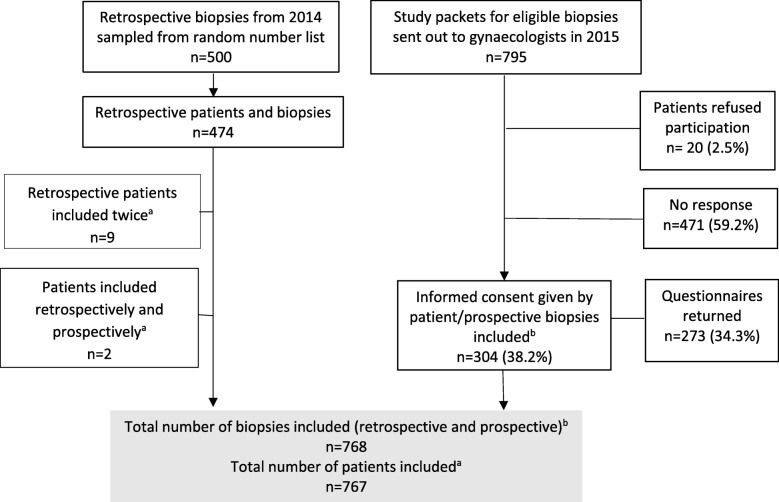
Study flow chart. Originally published in Swiss Federal Office of Public Health Bulletin (reference 19).^a^ 11 patients were included in the study twice by different laboratories.^b^ 10 biopsies were excluded because they were from the same patient with the same HPV result

We included a total of 768 biopsies from 767 women in the study (Fig. 1). For 11 women, two participating laboratories received a biopsy. Biopsies from the same patient with the same HPV results (*n* = 10) were considered only once in the analysis. One woman had two biopsies with different HPV results and both were included. From 2014, we retrospectively included 474 biopsies from 465 women. During 2015, we contacted the gynaecologists of 795 women diagnosed with CIN3+. Three hundred and four (38.2%) women gave informed consent, 20 declined participation and we received no reply from 471. In addition, 273 patients in 2015 completed and returned a patient questionnaire (Fig. 1).

The mean age of women included was 35.5 years (standard deviation, SD ±10.6, range 17–81). Seven-hundred and two of 768 (91.4%) biopsies were diagnosed with CIN3, 33 (4.3%) with adenocarcinoma in situ, 24 (3.1%) with squamous cell carcinoma and nine (1.2%) with adenocarcinoma. Characteristics of retrospective and prospective patients were similar (Additional file 1: Table S2). The diagnoses were based on the following samples: 424 (54.2%) cone biopsies, 292 (37.3%) punch biopsies, 24 (3.1%) curettage biopsies, 16 (2.0%) biopsies from hysterectomies and 27 (3.5%) biopsies from other types of procedures. Six of 273 (2.2%) of the women who completed the patient questionnaire reported that they have been diagnosed as HIV-positive and 9 (3.3%) women did not answer the question. Twenty-eight of 273 (10.3%) women who completed the patient questionnaire reported having received at least one dose of an HPV vaccine. Only one of 28 vaccinated women reported not having had sexual intercourse before receiving a single dose of quadrivalent vaccine. She had CIN3+ with HPV type 52. Twelve of the 28 women were > 26 years of age at the time of reported vaccination.

### HPV genotypes

Four hundred and seventy-five (61.8%; 95% CI 58.3–65.3) of the 768 biopsies were positive for HPV 16 only, HPV 18 only or both HPV 16 and 18. Four hundred and thirty five (56.6%; 95% CI 53.1–60.2) biopsies were positive for HPV 16 alone and 50 (6.5%; 95% CI 4.9–8.5) were positive for HPV 18 alone. One or more of the oncogenic HPV genotypes contained in the nonavalent HPV vaccine were present in 687 (89.5%; 95% CI 87.1–91.5) of the biopsies. One or more of the HPV genotypes classified as oncogenic by IARC were present in 727 (94.7%; 95% CI 92.8–96.1) of the biopsies. The HPV distribution for retrospectively and prospectively included biopsies was similar (Table 1).

**Table 1:** Oncogenic HPV genotypes present in CIN3 + plus study biopsies^a^

	Retrospective (2014)	Prospective (2015)	All	*p*-value
	Biopsies, *n* = 465	Biopsies, *n* = 303	*N* = 768	
	HPV types, n, % (95% CI)	HPV types, n, % (95% CI)		
HPV 16 and/ or 18	281, 60.4 (55.8–64.9)	194, 64.0 (58.3–69.4)	475, 61.8 (58.3–65.3)	0.316
Oncogenic HPV genotypes in nonavalent vaccine^b^	417, 89.7 (86.5–92.3)	270, 89.1 (85.0–92.4)	687, 89.5 (87.1–91.5)	0.802
All other high-risk HPV genotypes^c^	45, 9.7 (7.1–12.7)	21, 6.9 (4.3–10.4)	66, 8.6 (6.7–10.8)	0.184

Abbreviations: *CI* confidence interval

^a^Multiple HPV genotypes may be present in one biopsy so the total number of HPV types is higher than the number of biopsies

^b^One more of the following HPV genotypes present: 16, 18, 31, 33, 45, 52, 58

^c^One or more of the following HPV genotypes present: 35, 39, 51, 56, 59

Multiple HPV genotypes were detected in 95 (12.4%; 95% CI 10.1–14.9) of the tested biopsies. The ranking of the ten most frequent HPV genotypes in our samples was: HPV 16, 56.6%; HPV 31, 12.5%; HPV 33, 7.2%; HPV 18, 6.5%; HPV 52, 5.7%; HPV 58, 4.3%; HPV 51, 3.4%; HPV 35, 2.5%; HPV 45, 2.0%; HPV 42, 1.3% (Additional file 1: Table S3). The frequencies of HPV 16 and/or 18, HPV oncogenic genotypes contained in the nonavalent vaccine or HPV genotypes classified as oncogenic by IARC did not differ according to Swiss-SEP quintile level (*p* = 0.167).

HPV 16 only, HPV 18 only or both HPV 16 and 18 were present in 427/702 (60.8, 95% CI 57.1–64.5) of the biopsies diagnosed as CIN3 and 24/33 (72.7, 95% CI 54.5–86.7) of invasive cervical cancers (adenocarcinoma and squamous cell carcinoma). At least one of the HPV genotypes contained in the nonavalent vaccine was detected in 626/702 (89.2, 95% CI 86.6–91.4) of the biopsies diagnosed as CIN3 and 32/33 (97.0, 95% CI 84.2–99.9) of invasive cervical cancers (Table 2). The frequency of individual HPV genotypes according to histological diagnosis is shown in Additional file 1: Table S4.

**Table 2:** HPV genotype distribution according to histological diagnosis

HPV genotype^a^	CIN3	Adenocarcinoma in situ	Squamous cell carcinoma	Adenocarcinoma	Invasive cancers (Squamous cell and adenocarcinoma)
	*N* = 702	*N* = 33	*N* = 24	*N* = 9	N = 33
	n, % (95 CI)	n, %, (95 CI^b^)	n, %, (95 CI^b^)	n, %, (95 CI^b^)	n, %, (95 CI^b^)
HPV 16 and/or 18	427, 60.8 (57.1–64.5)	24, 72.7 (54.5–86.7)	16, 66.7 (44.7–84.4)	8, 88.9 (51.8–99.7)	24, 72.7 (54.5–86.7)
HVP 16 positive	398, 56.7 (52.9–60.4)	18, 54.5 (36.4–71.9)	14, 58.3 (36.6–77.9)	5, 55.6 (21.2–86.3)	19, 57.6 (39.2–74.5)
HPV 18 positive	36, 5.1 (3.6–7.0)	7, 21.2 (9.0–38.9)	3, 12.5 (2.7–32.4)	4, 44.4 (13.7–78.8)	7, 21.2 (9.0–38.9)
Any type in nonavalent vaccine^c^	626, 89.2 (86.6–91.4)	29, 87.9 (71.8–96.6)	23, 95.8 (78.9–99.9)	9, 100.0 (66.4–100.0)	32, 97.0 (84.2–99.9)
Any other IARC oncogenic type not in nonavalent vaccine^c^	56, 8.0 (6.1–10.2)	1, 3.0 (0.1–15.8)	1, 4.2 (0.1–21.1)	0, 0.0 (0.0–33.6)	1, 3.0 (0.1–15.8)
Any type not classified as oncogenic by IARC^d^	18, 2.6 (1.5–4.0)	0, 0.0 (0.0–10.6)	0, 0.0 (0.0–14.2)	0, 0.0 (0.0–33.6)	0, 0.0 (0.0–10.6)
HPV negative^e^	4, 0.6 (1.2–1.5)	1, 3.0 (0.1–15.8)	0, 0.0 (0.0–14.2)	0, 0.0 (0.0–33.6)	0, 0.0 (0.0–10.6)

*Abbreviations*: *CI* confidence interval, *IARC* International Agency for Research on Cancer

^a^Multiple HPV genotypes may be present for one biopsy, so column totals are greater than the number of biopsies and percentages do not sum to 100%

^b^A one-sided 97.5% confidence interval was calculated if there were zero observations

^c^Oncogenic HPV genotypes in the nonavalent vaccine: 16, 18, 31, 33, 45, 52, 58

^d^HPV genotypes classified as oncogenic by IARC: 16, 18, 31, 33, 35, 39, 45, 51, 52, 56, 58, 59

^e^After retesting by WHO Global HPV Reference Laboratory

### HPV negative and non-evaluable biopsies

On initial analysis, 729/768 (94.9%) samples were positive for HPV, 20 (2.6%) were negative for HPV and 19 (2.5%) were non-evaluable owing to the presence of PCR inhibitors or lack of material (Additional file 1: Figure S1). The WHO Global HPV Reference Laboratory in Stockholm, Sweden reanalysed 17 HPV negative samples and one non-evaluable biopsy with adequate material. Fifteen out of the 17 samples that were negative on original analysis were found to be positive for HPV. Two samples were confirmed as negative for HPV. Three of the negative samples were not confirmed because no material was available for retesting. One of the non-evaluable samples retested was found to be positive for HPV types 16, 18 and 51. In summary, 5/768 (0.7%; 95% CI 0.2–1.5) of the analysed samples were considered negative for HPV (Table 2) and 18/768 (2.3%; 95% CI 1.4–3.7) non-evaluable after confirmatory testing (Additional file 1: Figure S1).

### Comparison of the CIN3 + plus study group with women in the general population

We obtained a Swiss-SEP index for 748/767 (97.5%) of the enrolled women. The comparison of the CIN3+ plus study group with the SNC (*n* = 54,769) showed that both groups had the same Swiss-SEP index (mean = 64.6; *p* = 0.999). Fewer women in the CIN3+ plus study group were of Swiss nationality (63.2% vs. 69.6%; *p* = 0.024), more were born abroad (51.0% vs. 40.0%; *p* = 0.001) and more were single (48.9% vs. 45.5%; *p* < 0.001). Differences in proportions were also observed between the groups in regards to the canton of residence (*p* < 0.001) and highest education level obtained (*p* = 0.004) (Table 3).

**Table 3:** Demographic characteristics of women in the CIN3 + plus study group and age-adjusted Swiss National Cohort

	CIN3+ plus study group^a^ % or mean (95% CI)	Swiss National Cohort^b^ % or mean (95% CI)	*p*-value
Swiss-SEP Index	64.6 (63.8–65.4)	64.6 (64.5–64.7)	0.999
Canton of residence			
Zurich	27.5	35.2	< 0.001
Geneva	25.2	19.9	
Lucerne	15.5	18.6	
Ticino	14.0	15.8	
Basel-Land	9.7	6.0	
Basel-City	6.1	4.4	
Other & Unknown	2.1	0.0	
Country of birth			
Switzerland	49.0	60.0	0.001
Other	51.0	40.0	
Nationality			
Swiss	63.2	69.6	0.024
Other	36.8	30.4	
Civil status			
Single	48.9	45.5	< 0.001
Married	31.5	45.6	
Divorced	10.7	6.1	
Widowed	2.2	2.7	
Other	6.7	0.0	
Highest education level completed			
≤ Compulsory education	10.7	17.2	0.004
Upper secondary level	51.9	43.3	
Tertiary level	37.4	39.5	

*Abbreviations*: *CI* confidence interval, *SEP* socioeconomic position

^a^Numbers of observations available: Swiss-SEP Index, 748; canton of residence, 767; country of birth, 241; nationality, 266; civil status, 270; highest education level, 262

^b^Numbers of observations available: Swiss-SEP Index, 54769; canton of residence, 54769; country of birth, 54711; nationality, 54769; civil status, 54768; highest education level, 54769

Some factors associated with HPV infection differed between the CIN3 + plus study group and SHS (*n* = 3537) (Table 4). Women in the CIN3+ plus study were more likely to have reported ≥2 sexual partners in the last 12 months (15.4% vs. 6.8%; *p* = 0.001) and reported younger age at first sex (17.5 vs. 18.1 years; *p* = 0.005). Women in the CIN3+ plus study were more likely to report that they were smokers (38.5% vs. 25.5%; *p* < 0.001). The proportion of women who reported hormonal contraception use in the last 12 months was 35.5% in the CIN3+ plus group and 41.7% in the SHS group (*p* = 0.072). A higher proportion of women in the CIN3+ plus study group reported ever having a cervical cancer screening test (89.0% vs. 81.5%; *p* = 0.002).

**Table 4:** Factors associated with cervical cancer in CIN3+ plus study group compared with the Swiss Health Survey

	CIN3+ plus study group *n* = 273	Swiss Health Survey *n* = 3537	*p*-value
	% or mean (95% CI)	% or mean (95% CI)	
Number of sexual partners in the last 12 months			
0	9.2	13.8	< 0.001^a^
1	74.0	78.8	
≥ 2	15.4	6.8	
Unknown	0.0	0.0	
No answer	1.5	0.6	
Age at first sexual intercourse^b^	17.5 (17.2–17.9)	18.1 (17.9–18.2)	0.005
Reported hormonal contraception use in the last 12 months^c^	35.5	41.7	0.072
Current smoker	38.5	25.6	< 0.001
	61.5	74.4	
Have you ever had a Pap-test?^d^			
Yes	89.0	81.5	0.002^d^
No	8.8	16.3	
Unknown	1.1	0.5	
No answer	1.1	0.0	
Not asked	0.0	1.7	

*Abbreviations*: *CI* confidence interval

^a^Unknown, no answer and not asked categories were not included in statistical analysis

^b^The number of observations for age at first sex was 270 for the CIN3 + plus study group and 2914 for the Swiss Health Survey

^c^Includes any intrauterine device for the Swiss Health Survey

^d^Women were asked if they ever had a cervical cancer screening test before the start of their current illness for the CIN3 + plus study

## Discussion

In this cross-sectional study of women with CIN3+ in 2014 and 2015, HPV genotypes 16 and/or 18 were detected in 61.8% of the biopsies analysed and HPV genotypes 16, 18, 31, 33, 45, 52 and/or 58 contained in the nonavalent vaccine, were detected in 89.5% of biopsies. 84.9% contained a single HPV type, 12.4% contained multiple HPV types and only 0.7% of samples had no HPV detected. Only 10.3% of the women who completed a patient questionnaire reported receiving a HPV vaccine. Socioeconomic position was the same for women in the CIN3 + plus study group and the SNC. The CIN3+ plus study population consisted of more single, non-Swiss women born abroad when compared to the Swiss general population. The CIN3+ plus study group had a higher proportion of women with ≥2 partners in the last 12 months, current smokers and were younger at the age of first sexual intercourse when compared with the SHS.

### Strengths and weaknesses

A strength of our study for public health surveillance of vaccine impact is that we analysed whole tissue biopsies with CIN3+ rather than cytological samples from cervical smears. This outcome is closer to the clinical endpoint of interest, cervical cancer, than HPV infection or mild dysplasia, which often regress. Additional strengths include the enrolment of women from all three language regions in Switzerland, collection of demographic and behavioural data that allowed a comparison with women in the general population and the inclusion of public, private and university laboratories. Although we only conducted the CIN3 + plus study in six of the 26 Swiss cantons, these cantons include 38.4% of the entire Swiss female population (1612992) [26]. An important limitation of this study is that participating laboratories did not use a standardised test for HPV detection, which could have affected our results. Some HPV genotype testing methods have been shown to produce comparable results in FFPE [27, 28]. However, for many assays, especially laboratory developed tests, formal comparisons are lacking and only three out of the eight testing laboratories participated in the WHO HPV Laboratory Network proficiency panel testing programme. Variability in test technology is expected in decentralised public health surveillance systems. To ensure that we did not miss HPV infections in samples that tested negative, an independent laboratory retested all negative specimens with enough available material; the final proportion of specimens with no HPV detected was very low. Another limitation was the apparently low percentage of eligible women who completed the questionnaire. We do not believe that non-participation resulted in bias because very few women specifically declined participation, so it is likely that gynaecologists simply did not have time to explain the study and obtain consent. If a surveillance system were introduced, additional information would probably be limited to HPV vaccination status and informed consent would not be required.

### Comparison with other studies

We found few reports of studies conducted to provide baseline data for the monitoring of HPV vaccination impact. Dobec et al. examined specimens from 202 women in Switzerland with cytological abnormalities in 2007 [29]. High risk HPV types were found in 98 of 136 women with low-grade squamous intraepithelial lesions (72.1%; 95% CI 64.0–78.9) and 32 of 33 with high-grade squamous intraepithelial lesions identified by cytology (97.0%; 95% CI 83.4–99.9). There were no cases of invasive carcinoma in that study. One study in Australia examined the distribution of HPV genotypes in specimens from 847 women with invasive cancer from 2005 to 2015, before HPV vaccination (introduced in 2007) would be expected to have had an impact [10]. Brotherton and colleagues found 0.8% with multiple HPV genotypes and 7.1% samples with no HPV detected. These differences probably result from the different study populations, or from suboptimal DNA extraction at the deparaffinisation stage. We studied specimens from 2014 to 2015, of which 91.4% were CIN3. In small countries like Switzerland, the number of women with invasive cervical cancer is too low for this to be used as an outcome on its own. Between the years 2010–2014, only 1271 cases (approximately 250 cases/year) of cervical cancer were registered with the National Institute for Cancer Epidemiology and Registration [30].

The top ranking HPV genotypes found in our study are comparable to other European countries, although the relative importance varies, depending on the specimen type and histological diagnosis [31–36]. In the CIN3+ plus study, dominated by CIN3, the top five HPV genotypes were HPV 16, 31, 33, 18 and 52. In Europe, the top five ranking HPV genotypes in cytological and tissue samples with high grade cervical precancerous lesions are HPV 16, 31, 33, 52 and 18 [37]. In invasive cervical cancer, HPV genotypes 16, 18, 33, 45 and 31 are the most frequent in cytological and tissue samples in Europe [38]. In CIN3+ plus, HPV types 16, 18, 33, 45 and 56 were the most frequent in invasive cancers, but there was only one biopsy with HPV 56 and none with HPV 31. A meta-analysis of studies that examined exclusively tissue samples with CIN3 worldwide, excluding Africa, Western/Central Asia and South/Central America, found a ranking with a prevalence of HPV 16, 54.5%; HPV 33, 11.0%; HPV 52, 10.9%; HPV 58, 10.8%, HPV 31, 10.7% [39]. Another meta-analysis examining women with invasive cervical cancer worldwide, found that after HPV genotypes 16 and 18, the most common genotypes (31, 33, 35, 45, 52 and 58) were the same in all continents, but with differences in frequencies [3]. All but three (35, 42 and 51) of the top 10 ranking HPV genotypes in our study are covered by the nonavalent vaccine.

### Interpretation of the study

In the CIN3+ plus study, 61.8% of the biopsies were positive for HPV genotypes 16 and/or 18 and could be covered by the use of bivalent and quadrivalent vaccines. Cross-protection against HPV genotypes 31, 33 and 45 provided by the bivalent or quadrivalent vaccine could increase this estimate [40]. Up to 89.5% of biopsies analysed in this study contain HPV genotypes in the nonavalent vaccine. The introduction of the nonavalent vaccine in Switzerland could help prevent almost all cases of CIN3+ if HPV vaccine uptake is adequate. In 2014–2016, the Swiss National Vaccination Coverage Surveys calculated that 47.7% (95% CI 40.8–54.6%) of 16 year old girls had received two doses of a HPV vaccine. Cantons with school-based HPV programs had a vaccination rate of 50.6% (95% CI 47.8–53.5%), whereas cantons without a school-based program had a lower rate of 37.2% (95% CI 34.1–40.3%) [41]. In countries like Australia and England, more than 70% of young women are vaccinated and steep reductions in genital warts and HPV genotypes following use of the quadrivalent vaccine in Australia suggest that this level of coverage provides herd protection [42–44]. A study in one canton in Switzerland with 70% HPV vaccine uptake also found a reduction in these HPV genotypes in self-collected cervicovaginal samples in the youngest women participating in cervical cancer screening 5 years after vaccine implementation [45], whilst a modelling study suggests that the current average level of HPV vaccination in Switzerland would also be sufficient to reduce the prevalence of HPV16 [46]. The data collected in the CIN3+ plus study reflect HPV genotypes at the start of the cantonal vaccination programmes. Only 10.3% of the women who completed a patient questionnaire reported receiving a HPV vaccine. Only one of 28 vaccinated women reported having received HPV vaccine before the onset of sexual intercourse and had a lesion with an HPV genotype that was not in the vaccine that she received. Therefore, it is highly likely that most of the vaccinated women were already infected with HPV at the time of vaccination.

When compared with the Swiss general population, women in the CIN3+ plus study group were more likely to have been born abroad, be single and to have completed secondary level education. The higher proportion of non-Swiss in our study population could be due to low screening rates in this population before arrival to Switzerland. These observed differences could also be due to the canton of residence and reflect regional differences. These results are partially consistent with a study analysing data trends from 1992 to 2012 in Switzerland that found that single women, those with a lower education level and who were non-Swiss were less likely to receive cervical cancer screening [47]. The Swiss-SEP index was, however, similar in women in the CIN3+ plus study and women in the Swiss general population. This finding contrasts with the results of many studies that have found that women with lower socioeconomic position are more likely to be diagnosed with cervical cancer owing to lower levels of cytological screening [48]. Possible explanations for our findings in Switzerland are that the Swiss-SEP does not capture relevant characteristics, that the proportion of Swiss women below the poverty line is low (7.6%) [49], or that the Swiss law requiring mandatory medical insurance provides universal health coverage [50]. Women in the CIN3+ plus study group were more likely to smoke and had higher numbers of recent sexual partners than women in the general population participating in the SHS, but were less likely to use hormonal contraception. Our results confirm the findings of other studies with regard to smoking. Factors that are known or thought to be associated with severe HPV-related disease include number of lifetime partners, oral contraception use and smoking [51]. Associations between hormone use and cervical neoplasia are inconsistent [52].

### Implications for public health and for future research

Monitoring of CIN3+ lesions, which are the clinical endpoints of interest, will be particularly important in countries with sub-optimal HPV vaccine coverage in which vaccination herd effects are less likely. The data from this study also suggest that introduction of the nonavalent HPV vaccine could help to prevent most CIN3+ cases in Switzerland if the uptake of HPV vaccination can increase further. The introduction of a two-dose vaccine schedule in 2012 for girls 11–14 years old and for boys since 2015 [53] should help to increase HPV vaccine coverage. In Switzerland, the cantonal cancer registries record cervical cancers and most of them have also collected data on CIN3 for several years. A new Swiss law on cancer registration will make data collection about cervical cancer and CIN3 mandatory, so the completeness of surveillance will increase.

The results of this study provide valuable information for the establishment of a surveillance system for HPV-associated cancers, especially cervical cancer, in Switzerland and other countries with similar healthcare systems [54]. Monitoring the changes in HPV genotype distribution will allow ongoing assessment of the impact of HPV vaccination and about potential, but unlikely, type replacement. HPV genotyping could be conducted on FFPE biopsies as conducted in this study or could be conducted on liquid- based cytology before histopathology, although differences in HPV genotype distribution may be obtained due to mixing of normal and lesion regions [55]. The six cantons and 10 pathology institutes that took part in the CIN3+ plus study could become a sentinel surveillance system, rather than requiring all 26 Swiss cantons to take part, since the characteristics of these cantons (Additional file 1: Table S5) and the distribution of HPV vaccination uptake is broadly similar to that of the whole country [41]. Statistical weighting of data from sentinel cantons can be applied to achieve a sample that represents the national distribution of characteristics such as language region. Laboratories involved in a surveillance programmes should participate in a quality assurance programme for HPV genotyping.

## Conclusions

In conclusion, we have documented the HPV type distribution in high-grade cervical lesions at the beginning of the Swiss cantonal HPV vaccination programmes. Quadrivalent and bivalent vaccines cover around 62% of CIN3+ lesions and 73% of invasive cervical cancers, whereas the nonavalent vaccine would cover about 90% of CIN3+ lesions and 97% of invasive cervical cancers. Together, sentinel surveillance of HPV genotype distribution in CIN3+ lesions, including information about HPV vaccination, and of cervical cancer incidence in cancer registries will provide the information needed to monitor HPV vaccine effectiveness in Switzerland and to plan the required public health activities.

## Additional files

Additional file 1: **Table S1.** Primary tests used by laboratories and HPV genotypes included in test. **Table S2.** Comparison of retrospective and prospective patient characteristics. **Table S3.** HPV genotype distribution according to rank after confirmatory testing by WHO HPV Reference Laboratory (*n* = 768). **Table S4.** HPV genotype distribution according to histological diagnosis. **Table S5.** Comparison of female general population in Switzerland and female population in CIN3+plus study cantons in 2016. **Figure S1.** Retesting of HPV negative and non-evaluable biopsies by WHO HPV Reference Laboratory. (DOCX 70 kb)

## Abbreviations

CI: Confidence interval
CIN: Cervical intraepithelial neoplasia
CIN3+: Cervical intraepithelial neoplasia or worse
DNA: Deoxyribonucleic acid
ECDC: European Centre for Disease Prevention and Control
FFPE: Formaldehyde-fixed paraffin-embedded
HPV: Human papillomavirus
IARC: International Agency for Cancer Research
MGP-PCR: Modified general primer-polymerase chain reaction
SHS: Swiss Health Survey
SNC: Swiss National Cohort
Swiss-SEP: Swiss index of socioeconomic position
WHO: World Health Organization
