# Supplementary material for: Human papillomavirus genotype distribution and socio-behavioural characteristics in women with cervical pre-cancer and cancer at the start of a human papillomavirus vaccination programme: the CIN3+ plus study
Source: BMC Cancer. 2019 Jan 30;19:111. doi: 10.1186/s12885-018-5248-y (PMC6354352; doi:10.1186/s12885-018-5248-y)
Supplement: Supplementary file 1 — Table S1. Primary tests used by laboratories and HPV genotypes included in test. Table S2. Comparison of retrospective and prospective patient characteristics. Table S3. HPV genotype distribution according to rank after confirmatory testing by WHO HPV Reference Laboratory (n = 768). Table S4. HPV genotype distribution according to histological diagnosis. Table S5. Comparison of female general population in Switzerland and female population in CIN3+plus study cantons in 2016. Figure S1. Retesting of HPV negative and non-evaluable biopsies by WHO HPV Reference Laboratory. (DOCX 70 kb) [file 12885_2018_5248_MOESM1_ESM.docx]

**Human papillomavirus genotype distribution and socio-behavioural characteristics in women with cervical pre-cancer and cancer at the start of a human papillomavirus vaccination programme: the CIN3+plus study**

Dianne Egli-Gany^1*^, Anne Spaar Zographos^2*^, Joachim Diebold^3^, Virginie Masserey Spicher^2^, Brigitte Frey Tirri^4^, Rolf Heusser^5^, Joakim Dillner^6^, Patrick Petignat^7^, Roland Sahli^8^, Nicola Low^1^ on behalf of the CIN3+plus study group

^1^Institute of Social and Preventive Medicine, University of Bern, Switzerland

^2^Federal Office of Public Health, Switzerland

^3^Institute of Pathology, Cantonal Hospital Lucerne, Switzerland

^4^Department of Gynaecology and Obstetrics, Cantonal Hospital Baselland, Switzerland

^5^National Institute for Cancer Epidemiology and Registration, Switzerland

^6^International HPV Reference Laboratory, Sweden

^7^Department of Gynaecology and Obstetrics, Geneva University Hospitals, Switzerland

^8^Institute of Microbiology, Lausanne University Hospital and University of Lausanne, Switzerland

*These authors contributed equally to this work

**Table of Contents**

[Supplementary Table 1. Primary tests used by laboratories and HPV genotypes included in test 2](#_Toc521521385)

[Supplementary Table 2. Comparison of retrospective and prospective patient characteristics 3](#_Toc521521386)

[Supplementary Table 3. HPV genotype distribution according to rank after confirmatory testing by WHO HPV Reference Laboratory (n=768) 4](#_Toc521521387)

[Supplementary Table 4. HPV genotype distribution according to histological diagnosis 5](#_Toc521521388)

[Supplementary Table 5. Comparison of female general population in Switzerland and female population in CIN3+plus study cantons in 2016 6](#_Toc521521389)

[Supplementary Figure 1. Retesting of HPV negative and non-evaluable biopsies by WHO HPV Reference Laboratory 7](#_Toc521521390)

# **Table S1. Primary tests used by laboratories and HPV genotypes included in test**

| Test name; Company name, location | Primary test at laboratory (n) | Nonavalent genotypes included^a^ | High-risk genotypes included^b^ | Other HPV genotypes included in test |
| --- | --- | --- | --- | --- |
| Line probe assay, INNO-LiPA™; Innogenetics N.V., Ghent, Belgium | 1 | yes | yes | 26, 40, 43, 44, 53, 54, 66, 68, 69, 70, 71, 73, 74, 82 |
| Laboratory developed test (1) | 1 | yes | yes | 1, 2, 7, 10, 13, 26, 28, 32, 40, 42, 43, 44, 53, 54, 55, 57, 61, 62, 66, 67, 68, 69, 70, 72, 73, 74, 81, 82, 83, 84, 86, 87, 89, 90, 91 |
| Laboratory developed test (2) | 1 | yes | yes | All genotypes are covered |
| Linear-Array™;  Roche Diagnostics, Rotkreuz, Switzerland | 1 | yes | yes | 26, 40, 42, 53, 54, 55, 61, 62, 64, 66, 67, 68, 69, 70, 71, 72, 73, 81, 82, 83, 84 |
| PGMY and MGR primer sets | 1 | yes | yes | 26, 40, 42, 53, 54, 55, 61, 62, 64, 66, 67, 68, 69, 70,71, 72, 73, 81, 82, 83, 84 |
| Seegene Anyplex II™; Seegene, Seoul, South Korea | 3 | yes | yes | 26, 40, 42, 43, 44, 53, 54, 61, 66, 68, 69, 70, 73, 82 |

^a^ HPV genotypes in the nonavalent vaccine: 6, 11, 16, 18, 31, 33, 45, 52, 58;

^b^ Oncogenic HPV genotypes according to the International Agency for Research on Cancer (2012): 16, 18, 31, 33, 35, 39, 45, 51, 52, 56, 58, 59.

# **Table S2. Comparison of retrospective and prospective patient characteristics**

|  | Retrospective patients^b^  n (%) | Prospective patients^c^  n/n (%) | p-value |
| --- | --- | --- | --- |
| Age in years (n, mean ± SD) | 35.3±10.7 | 35.9±10.5 | 0.428 |
| Swiss-SEP (n, mean ± SD) | 64.5±11.2 | 64.8±10.2 | 0.682 |
| Diagnosis  CIN  Squamous cell carcinoma  Adenocarcinoma in situ  Adenocarcinoma | 426 (91.6)  16 (3.4)  18 (3.9)  5 (1.1) | 276 (91.1)  8 (2.6)  15 (5.0)  4 (1.3) | 0.808 |
| Canton  Zurich  Geneva  Lucerne  Ticino  Basel-Land  Basel-City  Other& Unknown | 134 (28.9)  132 (28.5)  63 (13.6)  63 (13.6)  34 (7.3)  29 (6.3)  8 (1.7) | 77 (25.3)  61 (20.1)  56 (18.4)  44 (14.5)  40 (13.2)  18 (5.9)  8 (2.6) | 0.015 |

^a^ Abbreviations: CIN, Cervical intraepithelial neoplasia; SD, standard deviation; SEP, socioeconomic position;

^b^ Number of observations available: Age, 430; Swiss-SEP, 456; diagnosis, 465; canton, 463;

^c^ Number of observations available: Age, 291; Swiss-SEP, 292; diagnosis, 303; canton, 304.

# **Table S3. HPV genotype distribution according to rank after confirmatory testing by WHO HPV Reference Laboratory (n=768)**

| Rank | HPV genotype^a^ | Frequency, n | % (95% CI) |
| --- | --- | --- | --- |
| 1 | 16^b^ | 435 | 56.6 (53.1-60.2) |
| 2 | 31^b^ | 96 | 12.5 (10.2-15.0) |
| 3 | 33^b^ | 55 | 7.2 (5.4-9.2) |
| 4 | 18^b^ | 50 | 6.5 (4.9-8.5) |
| 5 | 52^b^ | 44 | 5.7 (4.2-7.6) |
| 6 | 58^b^ | 33 | 4.3 (3.0-6.0) |
| 7 | 51^b^ | 26 | 3.4 (2.2-4.9) |
| 8 | 35^b^ | 19 | 2.5 (1.5-3.8) |
| 9 | 45^b^ | 15 | 2.0 (1.1-3.2) |
| 10 | 42 | 10 | 1.3 (0.6-2.4) |
| 11 | 56^b^ | 9 | 1.2 (0.5-2.2) |
| 12 | 39^b^ | 8 | 1.0 (0.5-2.0) |
| 12 | 73 | 8 | 1.0 (0.5-2.0) |
| 13 | 53 | 7 | 0.9 (0.4-1.9) |
| 13 | 82 | 7 | 0.9 (0.4-1.9) |
| 14 | 6 | 6 | 0.8 (0.3-1.7) |
| 14 | 66 | 6 | 0.8 (0.3-1.7) |
| 14 | 70 | 6 | 0.8 (0.3-1.7) |
| 14 | 59^b^ | 6 | 0.8 (0.3-1.7) |
| 15 | 68 | 5 | 0.7 (0.2-1.5) |
| 16 | 54 | 4 | 0.5 (0.1-1.3) |
| 17 | 44 | 3 | 0.4 (0.1-1.1) |
| 18 | 40 | 2 | 0.3 (0.0-0.9) |
| 19 | 43 | 1 | 0.1 (0.0-0.7) |
| 19 | 61 | 1 | 0.1 (0.0-0.7) |
| 19 | 89 | 1 | 0.1 (0.0-0.7) |
| not applicable | not evaluable | 18 | 2.3 (1.4-3.7) |
| not applicable | negative^c^ | 5 | 0.7 (0.2-1.5) |

^a^ Multiple HPV genotypes may be present for one biopsy, so totals are higher than the number of biopsies and percentages do not add to 100%;

^b^ HPV oncogenic genotypes according to the International Agency for Research on Cancer (2012) [2];

^c^ One HPV negative specimen was tested a third time and was found to be positive for HPV 73 and is reported as negative here.

# **Table S4. HPV genotype distribution according to histological diagnosis**

| HPV genotype^a^ | CIN3  N=702 | Adenocarcinoma in situ  N=33 | Squamous cell carcinoma  N=24 | Adenocarcinoma  N=9 | Invasive cancers (Squamous cell and adenocarcinoma)  N=33 |
| --- | --- | --- | --- | --- | --- |
|  | n (%) | n (%) | n (%) | n (%) | n (%) |
| HPV 16 | 398 (56.7) | 18 (54.5) | 14 (58.3) | 5 (55.6) | 19 (57.6) |
| HPV 18 | 36 (5.1) | 7 (21.2) | 3 (12.5) | 4 (44.4) | 7 (21.2) |
| HPV 31 | 94 (13.4) | 2 (6.1) | 0 (0.0) | 0 (0.0) | 0 (0.0) |
| HPV 33 | 47 (6.7) | 3 (9.1) | 4 (16.7) | 1 (11.1) | 5 (15.2) |
| HPV 35 | 18 (2.6) | 1(3.0) | 0 (0.0) | 0 (0.0) | 0 (0.0) |
| HPV 39 | 7 (1.0) | 1 (3.0) | 0 (0.0) | 0 (0.0) | 0 (0.0) |
| HPV 45 | 12 (1.7) | 0 (0.0) | 3 (12.5) | 0 (0.0) | 3 (9.1) |
| HPV 51 | 25 (3.6) | 1 (3.0) | 0 (0.0) | 0 (0.0) | 0 (0.0) |
| HPV 52 | 43 (6.1) | 1 (3.0) | 0 (0.0) | 0 (0.0) | 0 (0.0) |
| HPV 56 | 7 (1.0) | 1 (3.0) | 1 (4.2) | 0 (0.0) | 1 (3.0) |
| HPV 58 | 33 (4.7) | 0 (0.0) | 0 (0.0) | 0 (0.0) | 0 (0.0) |
| HPV 59 | 6 (0.9) | 0 (0.0) | 0 (0.0) | 0 (0.0) | 0 (0.0) |
| HPV negative | 4 (0.6) | 1 (3.0) | 0 (0.0) | 0 (0.0) | 0 (0.0) |

Abbreviations: CI, confidence interval;

^a^ Multiple HPV genotypes may be present for one biopsy, so column totals are greater than the number of biopsies and percentages do not sum to 100%;

^b^ Oncogenic HPV genotypes in the nonavalent vaccine: 16, 18, 31, 33, 45, 52, 58.

# **Table S5. Comparison of female general population in Switzerland and female population in CIN3+plus study cantons in 2016**

|  | Swiss Population  n (%) | CIN3+plus study cantons^a^  n (%) |
| --- | --- | --- |
| Female Population^b^ | 4,275,489 | 1,640,407 |
| Region^c^  German speaking  French speaking  Italian speaking | 2,975,933 (69.6%)  1,116,966 (26.1%)  182,590 (4.3%) | 1,203,854 (73.4%)  253,963(15.5%)  182,590 (11.1%) |
| Nationality^b^  Swiss  Not Swiss | 3,261,194 (76.3%)  1,014,295 (23.7%) | 1,204,158 (73.4%)  436,249 (26.6%) |
| Country of Birth^b^  Switzerland  Not Switzerland | 2,985,905 (69.8%)  1,289,584 (30.2%) | 1,058,322 (64.5%)  582,085 (35.5%) |
| Civil Status^d^  Single  Married  Widowed  Divorced  No indication | 1,757,041 (41.1%)  1,783,460 (41.7%)  329,303 (7.7%)  405,199 (9.5%)  486 (<0.1%) | 691,662 (42.2%)  664,114 (40.5%)  121,825 (7.4%)  162,606 (9.9%)  200 (<0.1%) |

^a^ Basel-City, Basel-Land, Geneva, Lucerne, Ticino, Zurich;

^b^ Swiss Federal Statistical Office: <https://www.pxweb.bfs.admin.ch/pxweb/de/px-x-0103010000_102/px-x-0103010000_102/px-x-0103010000_102.px/table/tableViewLayout2/?rxid=76556c86-c409-4ba4-93b6-840836398118>;

^c^ German speaking cantons; Aargau, Appenzell Ausserrhoden, Appenzell Innerrhoden, Basel-City, Basel-Land, Bern, Glarus, Graubünden, Lucerne, Nidwalden, Obwalden, St. Gallen, Schaffhausen, Schwyz, Solothurn, Thurgau, Uri, Zug, Zurich; French speaking cantons, Fribourg, Geneva, Jura, Neuchâtel, Valais, Vaud; Italian speaking canton, Ticino;

^d^ Swiss Federal Statistical Office: <https://www.pxweb.bfs.admin.ch/pxweb/en/px-x-0102010000_103/px-x-0102010000_103/px-x-0102010000_103.px/table/tableViewLayout2/?rxid=430d7e44-8f64-405f-b08a-a84c6d1f87cf>.

Non-evaluable at initial analysis

n=19

HPV negative at initial analysis

n=20

Retested at WHO Reference Lab

n=1

Not enough material for restesting

n=3

Not enough material for restesting

n=18

Retested at WHO Reference Lab

n=17

HPV positive after restesting

n=1

HPV positive after restesting

n=15

HPV negative after retesting

n=2

Final result: HPV negative

n=5

Final result: Non-evaluable

n=18

# **Figure S1. Retesting of HPV negative and non-evaluable biopsies by WHO HPV Reference Laboratory**
